# Raised Trappin2/elafin Protein in Cervico-Vaginal Fluid Is a Potential Predictor of Cervical Shortening and Spontaneous Preterm Birth

**DOI:** 10.1371/journal.pone.0100771

**Published:** 2014-07-30

**Authors:** Danielle S. Abbott, Evonne C. Chin-Smith, Paul T. Seed, Manju Chandiramani, Andrew H. Shennan, Rachel M. Tribe

**Affiliations:** 1 Division of Women's Health, King's College London, Women's Health Academic Centre King's Health Partners, London, United Kingdom; 2 Parturition Research Group, Department of Surgery & Cancer, Imperial College London, London, United Kingdom; 3 Division of Women's Health, King's College London Women's Health Academic Centre KHP, St. Thomas' Hospital Campus, London, United Kingdom; Rush University, United States of America

## Abstract

Early spontaneous preterm birth is associated with inflammation/infection and shortening of the cervix. We hypothesised that cervico-vaginal production of trappin2/elafin (peptidase inhibitor 3) and cathelicidin antimicrobial peptide (cathelicidin), key components of the innate immune system, are altered in women who have a spontaneous preterm birth. The aim was to determine the relationship between cervico-vaginal fluid (CVF) trappin2/elafin and cathelicidin protein concentrations with cervical length in woman at risk of spontaneous preterm birth. Trappin2/elafin and cathelicidin were measured using ELISA in longitudinal CVF samples (taken between 13 to 30 weeks' gestation) from 74 asymptomatic high risk women (based on obstetric history) recruited prospectively. Thirty six women developed a short cervix (<25 mm) by 24 weeks' and 38 women did not. Women who developed a short cervix had 2.71 times higher concentrations of CVF trappin2/elafin from 14 weeks' *versus* those who did not (CI 1.94–3.79, p<0.0005). CVF trappin2/elafin before 24 weeks' was 1.79 times higher in women who had a spontaneous preterm birth <37 weeks' (CI: 1.05–3.05, p = 0.034). Trappin2/elafin (>200 ng/ml) measured between 14^+0^–14^+6^ weeks' of pregnancy predicted women who subsequently developed a short cervix (n = 11, ROC area = 1.00, p = 0.008) within 8 weeks. Cathelicidin was not predictive of spontaneous delivery. Vitamin D status did not correlate with CVF antimicrobial peptide concentrations. Raised CVF trappin2/elafin has potential as an early pregnancy test for prediction of cervical shortening and spontaneous preterm birth. This justifies validation in a larger cohort.

## Introduction

Preterm birth is a global healthcare problem associated with significant neonatal morbidity and mortality and substantial healthcare costs [1]–[2]. Spontaneous preterm birth (sPTB) accounts for approximately three quarters of all premature deliveries and the need for early identification of at-risk women is widely recognised, since this would facilitate management and instigation of appropriate interventions. Current predictors commonly used in clinical practice to assess risk of sPTB include cervical length and cervico-vaginal fluid (CVF) fetal fibronectin (fFN), but their use is limited to gestational ages beyond 18 weeks' and positive predictive power is suboptimal [3]. Earlier and more accurate prediction of risk would be advantageous. A test which is safe, easy to perform and globally acceptable would also have applicability in low to middle income countries where the incidence of prematurity is high [4].

sPTB is closely linked with underlying inflammation and infection, and there has been considerable focus on the potential of inflammatory cytokines as predictive biomarkers [5]. However, few have questioned whether host defence peptides (antimicrobial peptides, AMPs;), key components of the innate immune defence system, might be alternative biomarkers for the same purpose [6]. Several families of AMPs (e.g. whey acidic proteins, trappin2/elafin, transferrins and human α and β defensins) have been identified in the female reproductive tract [7]–[9]. Trappin2/elafin (also known as peptidase inhibitor 3, PI3), a member of the whey acidic protein family, possesses anti-elastase and anti-protease 3 properties and exerts both antimicrobial and immunomodulatory actions at mucosal surfaces [6], [10]–[12]. The PI3 gene produces a spliced protein (117 aa; 12.3 kDa) which is cleaved intracellularly to a mature protein (9.9 kDA, Trappin 2). This can be secreted and tethered to the extracellular matrix via an exposed cementoin domain. Trappin2 can be further processed via extracellular tryptases to soluble elafin (6 kDA), a smaller molecule which is no longer tethered to the extracellular matrix [10]–[11].

Trappin2/elafin proteins are usually expressed constitutively at low concentrations within epithelial cell layers, but synthesis can be stimulated by lipopolysaccharide and inflammatory cytokines and down regulated by oestradiol [6], [10]–[11], [13]. PI3 mRNA and associated trappin2/elafin protein has been reported to be increased in the amnion of women delivering preterm with chorioamnionitis compared to those without, but conversely also found to be reduced in amnion from women with preterm premature rupture of the membranes (PPROM) [14]. Lower trappin2/elafin CVF concentrations are also reported in low risk pregnant women presenting with bacterial vaginosis [15]. Less is known about cathelicidin antimicrobial peptide (cathelicidin) in the human reproductive tract, but mRNA and protein have been detected in vaginal epithelium originating from non-pregnant women [16].

Our knowledge of the utility of CVF AMPs to predict sPTB is limited; the presence and gestational profiles of AMPs in CVF and their relation to other immune modulators such as inflammatory cytokines and vitamin D is not well described. This is despite growing evidence that inflammatory mediators modulate expression of AMPs and the recognition that vitamin D is integral to pathways regulating cathelicidin synthesis and metabolism [11], [15], [17], [18]. The relation between vitamin D and AMPs is of particular interest given reports suggesting a role for vitamin D insufficiency in poor pregnancy outcome [19], [20].

Trappin2/elafin has previously been identified as a potentially useful clinical biomarker of breast cancer [21], and in graft versus host disease of the skin following bone marrow transplantation [22]. It follows that better understanding of the role of trappin2/elafin and cathelicidin in the pathophysiology of spontaneous preterm birth may provide new avenues for prediction and treatment of women at high risk of spontaneous labour and early delivery.

We hypothesised that trappin2/elafin and cathelicidin concentrations in CVF would be altered in women at risk of sPTB. This study, therefore, has investigated the relationships between CVF trappin2/elafin and cathelicidin concentrations and cervical length in a cohort of woman at high risk of sPTB (based on obstetric history). The association between serum vitamin D concentration and trappin2/elafin and cathelicidin was also explored.

## Materials and Methods

### Ethics statement

Samples for AMP analysis were obtained from a subset of women recruited to a previously reported prospective observational study (the Cervical Length and Inflammatory Changes: CLIC study) [23] designed to assess the relationship between inflammation and cervical shortening in women at high risk (i.e. women with a previous history or late miscarriage or sPTB) of sPTB. This study was approved by the Research Ethics Committee of St Thomas' Hospital, London UK (06/Q0704/66). All patients provided written informed consent and self-reported information on ethnicity, which was classified for analysis purposes as ‘white, black or other’.

### Study population and design

Women were enrolled from two preterm surveillance clinics at two teaching hospitals in London between 13 and 24 weeks' gestation from June 2006 until November 2008. Women with a history of at least one prior spontaneous preterm birth or late miscarriage between 16 and 34 weeks' gestation were eligible to participate. Exclusion criteria were multiple pregnancy, previous iatrogenic preterm births and inability to give informed consent. Thereafter, recruits were assessed until 30 weeks' gestation with each providing a CVF sample prior to transvaginal cervical length assessment every two weeks.

However, if the cervical length shortened to less than 25 mm before 24 weeks' gestation, women (allocated to the case group) were offered treatment (either cervical cerclage or vaginal progesterone) according to clinical practice. In order to ensure equal numbers for analysis and to explore the impact of treatments on trappin2/elafin concentration, women were assigned to either treatment using computer generated open-label randomization by the study investigator. Samples and scans were then repeated weekly thereafter in women found to have a short cervix. The use of vaginally administered natural progesterone (Cyclogest, 400 mg once daily; Actavis UK Ltd, Devon, UK) was based on current clinical practice at the time of the study. Women who did not develop a short cervix by 24 weeks' gestation were allocated to the control group. Routine screening for vaginal organisms such as bacterial vaginosis, *Trichomonas vaginalis* and *Candida* was not included in the study protocol but if women presented with symptoms, a high vaginal swab was taken and treatment carried out according to antimicrobial sensitivities.

### Cervical length assessment

Cervical length was assessed in accordance with standardised guidelines [23]. In brief, a sagittal view of the cervix was obtained with the long axis view of the echogenic endocervical mucosa along the length of the canal, allowing identification of both the internal and external os. The linear distance between the external and internal os was recorded 3 times (in mm) over a minimum of 3 minutes using optimal magnification and zoom settings, and the shortest measurement recorded. Transfundal pressure was exerted for 15 seconds and subsequent demonstration of a funnel was noted. The total closed length in all women was measured and if a cerclage was present, the closed length cranial to the cerclage was also recorded.

### Sample preparation and AMP analysis

As reported previously [23], a single Dacron swab was obtained from the posterior vaginal fornix in order to obtain a high vaginal sample of CVF at each visit. During speculum examination, the swab was placed in the posterior vaginal fornix for 10 seconds to achieve saturation, then transferred into 750 µl of standard phosphate-buffered saline solution containing protease inhibitors (Complete, Roche Diagnostics GmbH, Germany) and immediately transported on ice to the laboratory. The swab was removed, placed in a clean tube, vortexed for 10 seconds and centrifuged (2600 g for 10 minutes at 4°C). Resultant fluid was collected and added to the fluid in the original tube. This was mixed and centrifuged for a further 10 minutes to remove cell debris. Cell-free supernatants were divided into aliquots (∼110 µl) and stored at ­80°C until analysis. Longitudinal trappin2/elafin CVF concentration analysis was undertaken on 437 individual samples from 74 women. Women from the control group were included for analysis if they had provided at least four longitudinal samples taken over the second trimester. A minimum of six samples were analysed for each woman who developed a short cervix (cases). Remaining samples from n = 64 women were used for cathelicidin analysis. The samples from cases included pre and post intervention samples, and the sample obtained at the visit when the cervix was found to be short prior to randomisation to an intervention. Samples were thawed at room temperature, briefly vortexed and analysed by ELISA [Trappin2/elafin, HK318; cathelicidin (LL-37), HK321 from Hycult, Biotech Cambridge] according to manufacturer's instructions. Samples used for trappin2/elafin measurement were diluted in sample buffer (1∶10 for control samples and 1∶50 or 1∶100 for case samples) to ensure positioning within the standard curve. CVF samples for cathelicidin measurement were undiluted. A pooled control sample achieved by combining a random set of 10 samples was included in individual plates to control for inter-plate variation. Final concentrations were calculated from the standard curves using logistic regression (Stata, Texas Version 11.2). For trappin2/elafin, the lower limit of detection of the ELISA was 0.878 ng/ml and maximal limit of detection was 10 ng/ml.

### Serum vitamin D analysis

Vitamin (25-OH)D concentrations were measured in all available serum samples (n = 67) taken at the first study visit using a chemiluminescent microparticle immunoassay (CMIA) for the quantitative determination of 25-hydroxyvitamin D (25-OH vitamin D) in human serum, according to manufacturer's instructions (ARCHITECT, Abbott Laboratories, Barcelona). For analysis, women were grouped into four categories of vitamin D status: <25 ng/ml; 25–49.9 ng/ml; 50–74.9 ng/ml and greater than 75 ng/ml.

### Statistical analysis

The sample size was not pre-determined due to inadequate published data informing the gestational profiles of CVF trappin2/elafin or cathelicidin concentrations. Rather, it was determined by the availability of CVF samples. This study and analysis was therefore exploratory in nature. Trappin2/elafin and cathelicidin concentrations were expressed as ng/ml. The study was not designed or powered to directly compare the two treatment groups or the relation between biochemical markers and spontaneous preterm birth, but some exploratory comparisons have been included.

Analysis was undertaken using Stata (version 11.2, Stata Corp, College Station, Texas). Distributions of data were first established by examination of distributional plots for raw and transformed values. Log transformations were applied to trappin2/elafin, cathelicidin and cytokine concentrations to achieve approximate Normality.

Where sample concentrations were below the limit of assay detection, an interval regression method was used, with the missing values taken as being at an unknown point on the interval between zero and the smallest positive concentration observed [24]. When considering multiple measurements from the same woman, a random effects regression model was used. In order to determine the difference in trappin2/elafin and cathelicidin expression in cases and controls prior to treatment, samples up to and including the visit at which the cervix shortened, but prior to treatment over a period of time from 13 to 24 weeks' were included. Post treatment samples were taken up to 30 weeks'. The average difference between cases and controls was determined using a regression model with correction for effect of gestation (2 weekly categories) and interassay plate variation. Adjustments for body mass index (BMI), maternal age and ethnicity, and current smoking status were considered. Results were expressed as ratios of the concentration, and as weekly rates of change as appropriate, with 95% confidence intervals. Actual p-values are given (usually to 2 decimal places), except for very small values, shown as p<0.001.

Spearman's rank correlations (r_s_) were used to show general association between markers. Graphs show geometric mean concentrations on a log scale, with standard error bars. Some descriptive data is provided as medians with quartiles. Test performance was described for prediction of sPTB and shortening cervix (prior to cervical shortening) using receiver operating characteristic (ROC) curves, sensitivity, specificity and predictive values.

## Results

One hundred and twelve women were enrolled into the CLIC observational study [23]. Thirty eight controls and 36 cases provided suitable samples for longitudinal analysis of CVF trappin2/elafin. Cathelicidin was measured in a subgroup (n = 34 controls and n = 30 cases) in whom there was sufficient CVF sample available. Table 1 summarises the baseline characteristics of the 74 women studied. The median (quartiles) gestational age of cervical shortening for cases (n = 36) was 19^+1.5^ weeks (17^+3^, 21^+2^).

**Table 1:** Demographics of women in study: high risk controls and cases (women who developed a short cervix before 24 weeks' gestation) providing cervico-vaginal swabs for serial trappin2/elafin measurements throughout gestation.

Characteristic	Sub category	Controls (n = 38)	Cases (n = 36)
Maternal age in years median (quartiles)		30.0 (22.4,35)	29.5 (27,34)
BMI in (kg/m^2^) at booking median (quartiles)		27.7 (23,32)	25.1 (23,28.6)
Risk Factors (N%)	previous PTB (24–34 weeks)	20 (53)	11 (31)
	previous PPROM	21 (55)	20 (56
	previous 2^nd^ trimester loss (16–24 weeks)	20 (53)	29 (81)
Current smoker N (%)		3 (8)	2 (6)
Ethnic group N (%)	White	12 (32)	4 (11)
	Black	23 (60)	30 (83)
	Other	3 (8)	2 (6)

Women in the control group had numerically more previous preterm deliveries between 24 and 34 weeks' gestation and were more likely to be white compared to women destined to develop a short cervix (<25 mm at <24 weeks' gestation), who reported more previous second trimester miscarriage and were more likely to be black. The incidence of bacterial vaginosis was similar between groups (controls, 16% and cases 14%). The sPTB <37 weeks'/late miscarriage rate was 16% for controls (n = 6 of 38 women, with one late miscarriage <24 weeks) and 53% for cases (short cervix group, n = 19 of 36 women, 8 of which were <24 weeks'). The demographics and sPTB rates were similar for the subgroup that provided samples for cathelicidin measurements.

### Trappin2/elafin gestational profile

**Figure 1:**
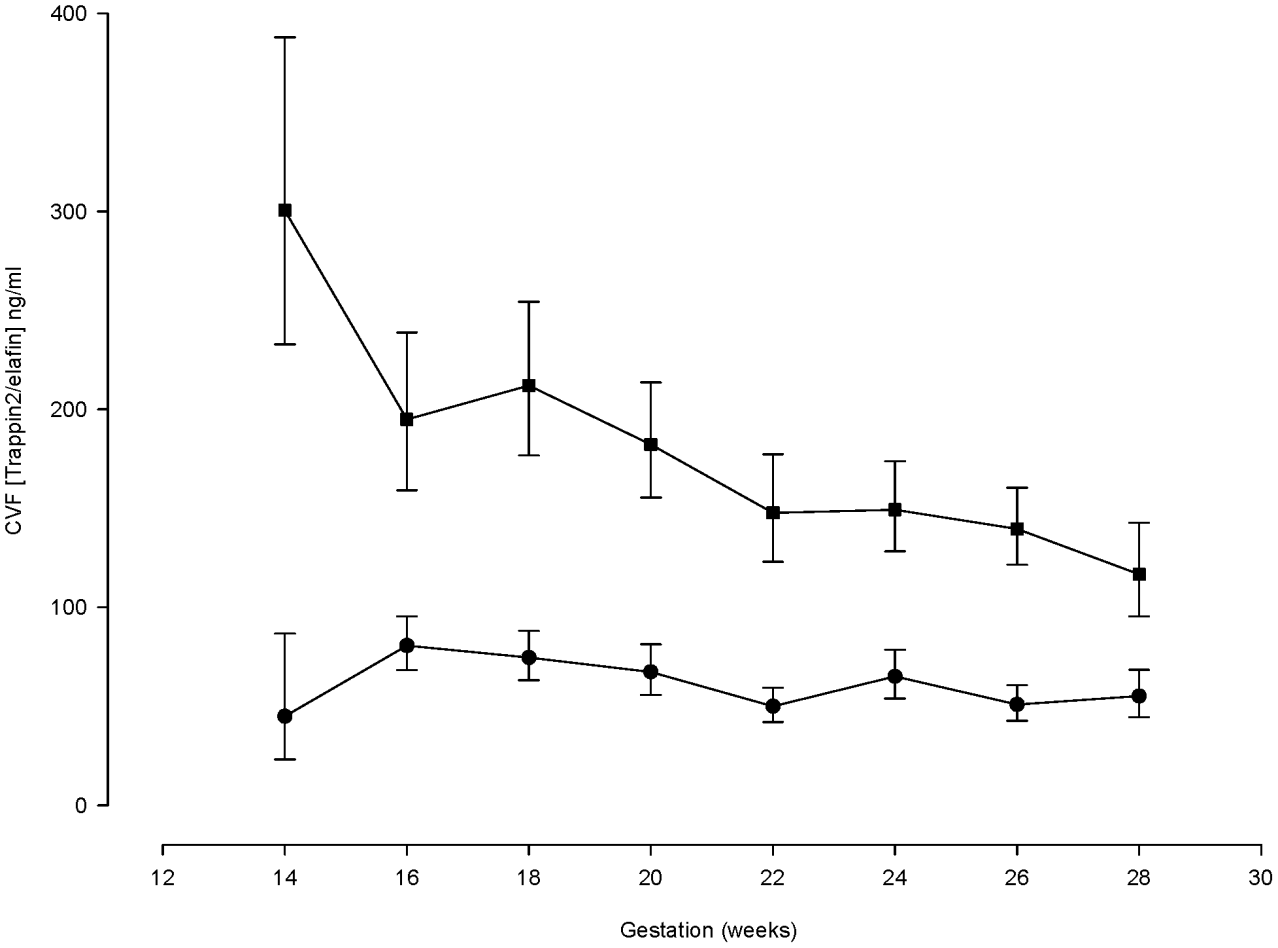
Longitudinal measurements of cervico-vaginal fluid (CVF) trappin2/elafin concentrations in women at high risk of spontaneous preterm birth who develop a short cervix compared to high-risk controls. The top (square symbol) line represents measurements in women who subsequently developed a short cervix (n = 36). The lower (circle symbol) line represents women in the high-risk control group who did not develop a short cervix (n = 38).

The CVF trappin2/elafin concentration was higher in women who subsequently developed a short cervix compared to high-risk controls with normal cervical length (Figure 1, ratio 2.71, CI 1.94 to 3.79, p<0.0005) and was maintained across the gestational range studied. Trappin2/elafin CVF concentrations were 3 fold higher than controls when cervical shortening was first detected (ratio 3.03 CI 1.92 to 4.81, p<0.0005).

The CVF trappin2/elafin concentration in women with a short cervix at the time of randomisation (prior to start of treatment) showed a non-significant trend towards reduction when measured four weeks later in the cerclage group, (ratio: 0.57 CI 0.32 to 1.02, p = 0.058). Trappin2/elafin was unaffected following four weeks of vaginal progesterone therapy (ratio: 1.05 CI 0.68 to 1.61, p = 0.830). Controls showed little gestation related change over a similar period. Trappin2/elafin measured before 24 weeks' was also higher in women who delivered following sPTB at <37 weeks' compared to those who delivered at term (ratio 1.79, CI: 1.05 to 3.05, p = 0.034). The influence of maternal ethnicity, BMI, age and current smoking status were also investigated as potential determinants of CVF trappin2/elafin concentrations, but no associations were found. As a result, these factors were not included in the final regression model.

### CVF trappin2/elafin as a predictor of a short cervix by 24 weeks and of sPTB

**Figure 2:**
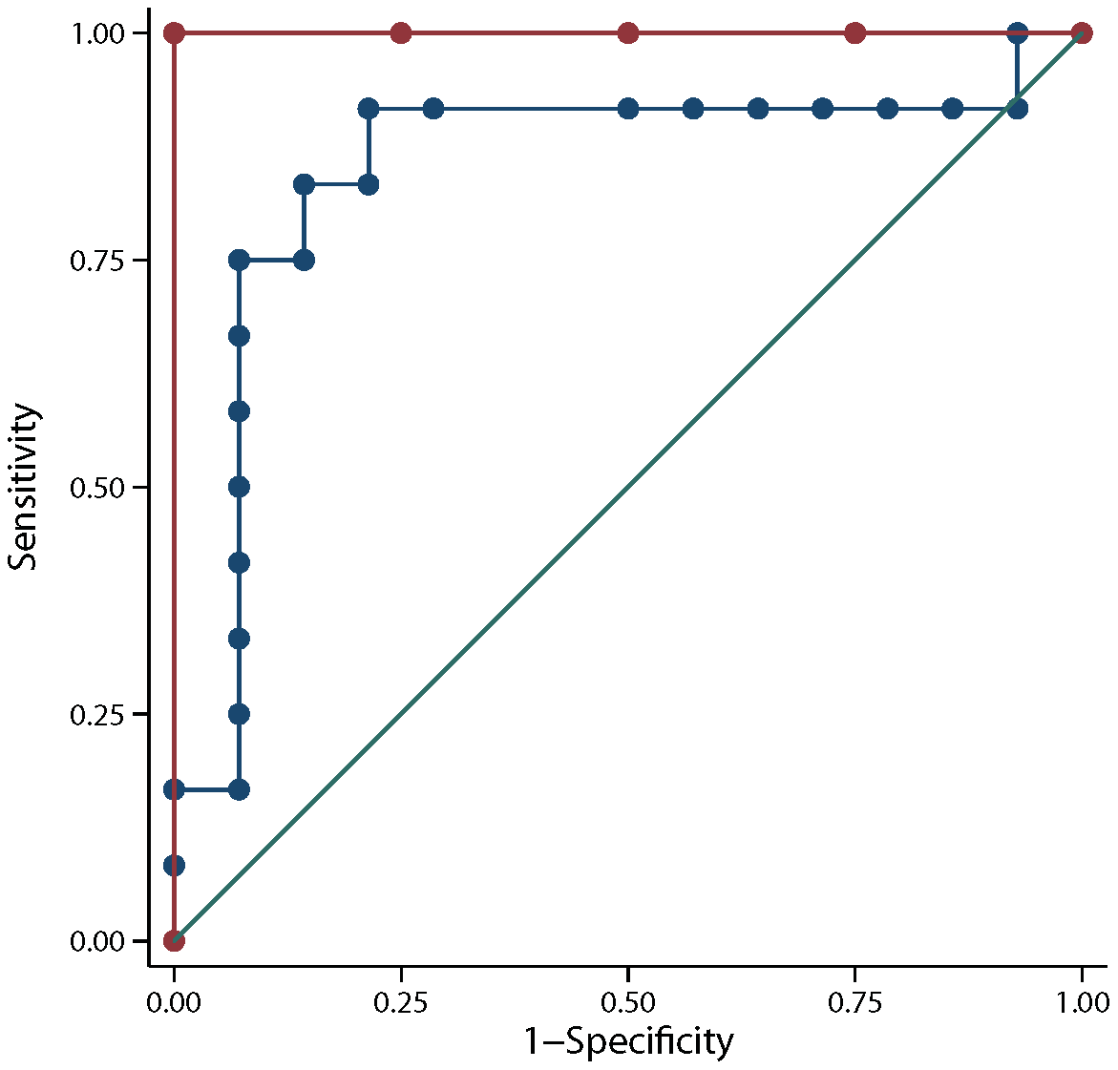
Receiver operating characteristic (ROC) curves demonstrating the ability of trappin2/elafin cervico-vaginal fluid values >200 pg/ml taken at 14–14^+6^ weeks' (red line, n = 11) and 14–15^+6^ (blue line, n = 26) to predict cervical shortening within ≤ 8 weeks in pregnant women at high risk of spontaneous preterm birth.

CVF trappin2/elafin measured between 14^+0^–14^+6^ weeks of pregnancy demonstrated a ROC area of 1.00 (n = 11, p = 0.008) for prediction of future cervical shortening in women with a normal cervical length (Figure 2). The areas under the ROC curve at 14^+0^ to 15^+6^, 16^+0^ to 17^+6^ and 18^+0^ to 19^+6^ weeks' were 0.85 (CI 0.67 to 1.00; n = 26), 0.76 (CI 0.59 to 0.92; n = 42) and 0.75 (CI 0.58 to 0.92; n = 41) respectively.

A concentration of >200 ng/ml provided the highest specificity for prediction of a short cervix by 24 weeks'. Performance of a single trappin2/elafin test, taken at 14^+0^ and 14^+6^ weeks' and the wider gestational window of 14^+0^ to 15^+6^ weeks, for prediction of a short cervix within 6 weeks and 8 weeks are shown in Table 2. For prediction of sPTB <37 weeks' gestation, the trappin2/elafin concentration at 14^+0^ to 15^+6^ weeks' was associated with an area under the ROC curve of 0.72 (CI 0.52 to 0.93; n  = 31).

**Table 2:** Prediction statistics for trappin2/elafin for values >200 ng/ml identifying high risk women who develop a short cervix within 6 or 8 weeks following the test.

Test Characteristics	14–14^+6^ weeks' (n = 11) to predict a short cervix at		14–15^+6^ weeks' (n = 26) to predict a short cervix at	
	≤6 weeks'	≤8 weeks'	≤6 weeks'	≤8 weeks'
Sensitivity (%)	100	100	80	83
Specificity (%)	80	100	81	83
Odds Ratio (%)	∞	∞	17.3	65.0
Positive Predictive Value (%)	86	100	73	91
Negative Predictive Value (%)	100	100	87	87

Test taken as a single measurement between either 14^+0^ to 14^+6^ or 14^+0^ to 15^+6^ weeks' in cases who developed a short cervix by 24 weeks'.

### Gestational cathelicidin profile

**Figure 3:**
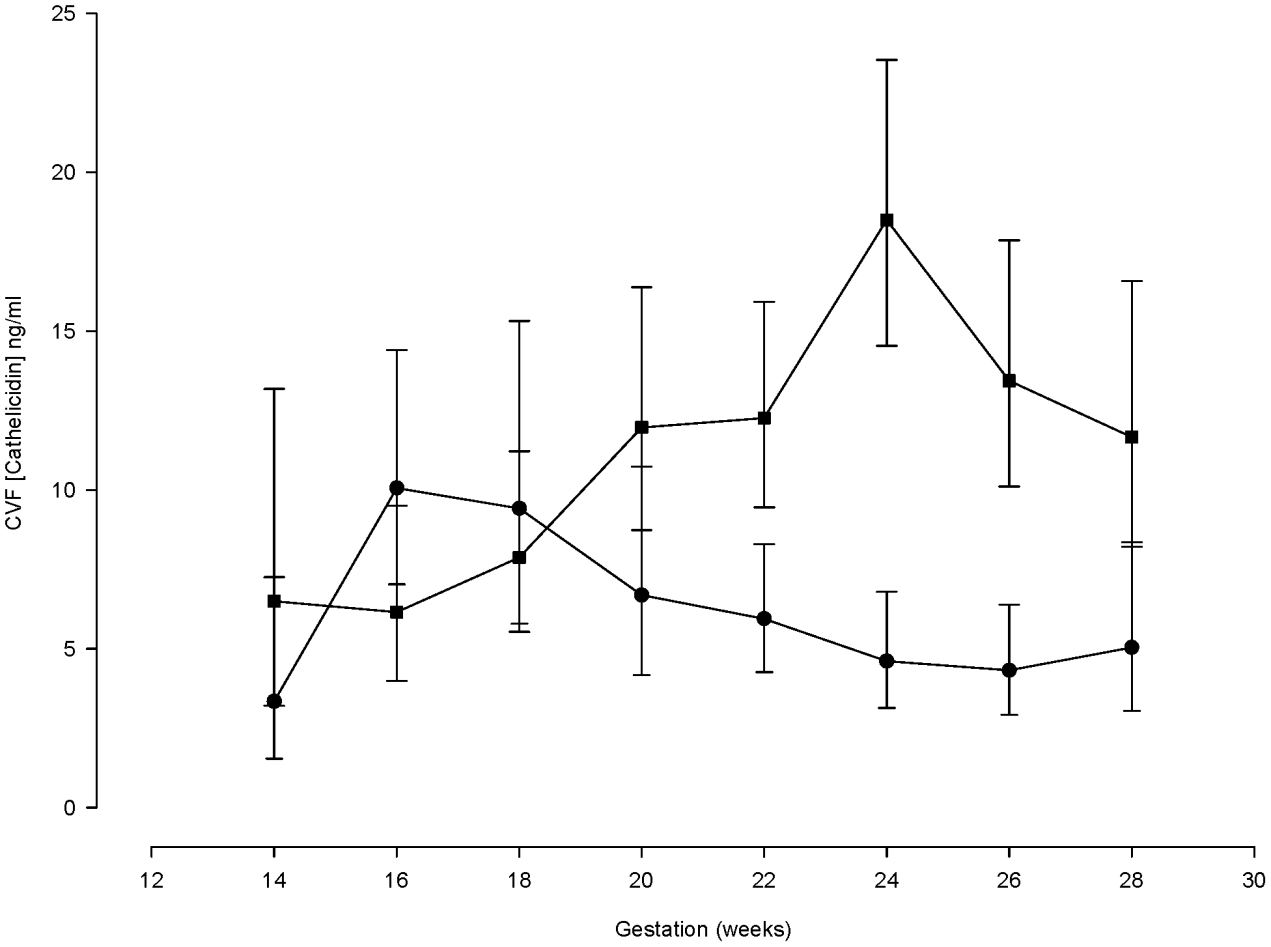
Longitudinal measurements of cervico-vaginal fluid cathelicidin concentrations in women at high risk of spontaneous preterm birth who develop a short cervix compared to high-risk controls. The square symbol line represents measurements in women who subsequently developed a short cervix (n = 30). The circle symbol line represents women in the high-risk control group who did not develop a short cervix (n = 34).

Cathelicidin concentrations (without adjustment for treatment or gestation) were similar in early pregnancy (14–18 weeks') in women who developed a short cervix by 24 weeks' (cases, mean gestation of cervical shortening 19^+2^ weeks') and high-risk controls (Figure 3). Subsequently, cathelicidin increased until 24 weeks', in contrast to controls which did not change. Overall, CVF cathelicidin rose by 12.7% (CI: 7.9 to 17.7; p = 0.008) per week in cases and fell by 5.6% (CI: 1.5 to 9.6) in controls (mean difference 19.41% (CI: 12.4 to 26.9, p<0.001) across the gestations studied.

In women with a short cervix, there was no significant influence of treatment (cerclage or vaginal progesterone) [P = 0.667]; the rate of rise in CVF cathelicidin concentrations across gestation was similar in women receiving progesterone [11.2% (CI: 2.9 to 20.1)] to those allocated to cerclage treatment [13.5% (CI: 7.7 to 19.6)]. No association between BMI, age or ethnicity and CVF cathelicidin concentrations were found.

### Prediction of a short cervix by 24 weeks' and sPTB using CVF cathelicidin measurements

CVF cathelicidin concentrations were not found to be predictive of a short cervix before 24 weeks' or for sPTB at <37 weeks of pregnancy (data not shown).

### Relation between CVF trappin2/elafin, cathelicidin and cytokines

There was no correlation between trappin2/elafin and any of the 11 cytokines measured [IL1B, IL4, IL6, IL7, colony stimulating factor 3 (granulocyte) (CSF3, previously G-CSF), colony stimulating factor 2 (CSF2, previously GM-CSF), interferon gamma (IFNG), chemokine (C-C motif) ligand 2 (CCL2, previously MCP-1), chemokine (C-C motif) ligand 4 (CCL4, previously referred to as MIP-1β) and tumour necrosis factor (TNF, previously TNF-alpha)]. A weak relationship with CCL2 was observed (r_s_ = 0.1729, p = 0.0025 after adjustment for multiple testing, n = 437).

In contrast, associations were found between cathelicidin and the cytokines measured (Table 3). For example, a doubling of IL1B concentrations in CVF was associated with an 88% increase in cathelicidin (P<0.001).

**Table 3:** Rank correlations between cathelicidin and 11 cytokines measured in matched cervico-vaginal fluid (CVF) samples (n = 167) taken between 13 and 24 weeks of gestation from 46 women.

Cytokine	Spearman correlations (r_s_) with CVF cathelicidin concentrations	P value
Interleukin 1B	0.6194	<0.001
Interleukin 4	0.5701	<0.001
Interleukin 6	0.4217	<0.001
Interleukin 7	0.5165	<0.001
Interleukin 8	0.5733	<0.001
Colony stimulating factor 3	0.4557	<0.001
Colony stimulating factor 2	0.5814	<0.001
Interferon gamma	0.4913	<0.001
Chemokine (C-C motif) ligand 2	0.4240	<0.001
Chemokine (C-C motif) ligand 4	0.4750	<0.001
Tumour necrosis factor (TNF)	0.5084	<0.001

### Vitamin (25-OH)D serum status in cases and controls

Serum vitamin (25-OH)D concentrations were measured at study entry in 35 women with a short cervix and 32 with a normal cervix (Table 4). Distribution between the four vitamin (OH-25)D categories was similar for both groups. The vitamin (OH-25)D concentration was not predictive of cervical shortening (area under ROC curve 0.52, CI: 0.38–0.67). There was no relationship between serum vitamin D, CVF trappin2/elafin or cathelicidin.

**Table 4:** Serum vitamin (OH-25) D levels in cases (with a short cervix) and controls (with a normal cervix).

Vitamin (25-OH) D category (ng/ml)	Cases (cervix <25 mm)	Controls (cervix >25 mm)
<25	8 (23%)	6 (19%)
25–49.9	21 (60%)	22 (69%)
50–74.9	4 (11%)	3 (9%)
>75	2 (6%)	1 (3%)
Total	35	32

## Discussion

Understanding of the relationships between cervico-vaginal host defence peptides and spontaneous preterm birth is limited. The novel demonstration that the CVF trappin2/elafin concentration is raised prior to cervical shortening in women at high risk of sPTB is indicative of an altered innate immune status early in pregnancy in these women. The substantive difference between cases and controls also suggests that measurement of trappin2/elafin may be a useful biomarker for identifying women at high risk of cervical shortening; i.e. those who could benefit from additional surveillance and intervention. In contrast, cathelicidin did not distinguish between high risk women who were likely to develop a short cervix and those who were not. The associations between cathelicidin and the cytokines measured suggest that cathelicidin is reflective of an inflammatory response following cervical shortening.

The reasons for raised CVF trappin2/elafin were not directly determined in this study. However, there is a wealth of literature describing the different roles trappin2/elafin proteins play in the innate immune response [6], [11], [25], [26]–[30]. Firstly, trappin2/elafin are well recognised as inhibitors of neutrophil elastase and protease 3 which are produced by activated neutrophils [6]. This is a protective response initiated by inflammatory mediators [11], [25] with trappin2/elafin proteins providing a ‘brake’ to excessive neutrophil activity and preventing damage of host tissues through induction of matrix metalloproteinases, glycoproteins, fibronectin and cadherins [26]–[28]. Neutrophils can also cause cleavage and release of elafin from trappin2. [29]. Secondly, trappin2/elafin can be induced directly in response to local infection, mediating disruption of bacterial membranes and inhibition of viral replication and/or attachment to epithelial cells [6], [30]. Thirdly, trappin2/elafin possesses immunomodulatory properties which generally promote Th1 cytokine production, neutrophil recruitment and NFκB activation in macrophages and dendritic cell activation [31]. Under some circumstances trappin2/elafin will also lead to suppression of cytokines dependent on the stage of the inflammatory process and disease context [31]. There is also potential for a genetic influence of trappin2/elafin synthesis as several PI3 polymorphisms have been identified [32], [33], but these are associated with a reduction in elafin expression.

This complexity of trappin2/elafin regulation provides a challenge to interpretation of data from the present study. Clearly pregnant women who develop a short cervix display produce more trappin2/elafin than high risk controls, and this may indeed reflect the ability to mount an enhanced host-defence response in response to unknown stimuli in the cervico-vaginal environment. Given the association between infection and sPTB, we would postulate that the underlying cause of raised CVF elafin in women with cervical shortening is the presence of a local subclinical bacteria/viral infection. In this scenario, trappin2/elafin would be induced via activation of pathogen recognition receptors (e.g. Toll-like receptors or RNA helicases) and downstream production of inflammatory cytokines, and possibly in response to escalating neutrophil elastase/protease 3 concentration(s). The observation that trappin2/elafin is raised throughout gestation indicates the stimulus persists during pregnancy, but that the innate response involving trappin2/elafin is not sufficient to inhibit cervical shortening or sPTB. CVF neutrophil elastase concentrations were not measured, but the potential recruitment of neutrophils, and release of neutrophil elastase in this scenario (providing a stimulus to increased CVF trappin2/elafin) provides a compelling explanation as to the mechanism which initiates cervical tissue damage, leading to shortening.

This hypothesis requires further investigation, as do alternative explanations such that women at risk of sPTB in this cohort may i) have constitutively raised trappin2/elafin basal production due to genetic predisposition or exposure to other stimuli or different reproductive tract microbiota profiles.

The lack of strong relationship between trappin2/elafin and CVF cytokine profiles is intriguing given that cytokines such as IL1B can, in the absence of pathogens, induce trappin2/elafin expression *in vitro*. Cytokines were detectable in CVF from women in the short cervix and control groups in this cohort, but only GM-CSF and MCP-1 [23] related to cervical shortening and these showed no correlation with trappin2/elafin. Possibly, as a result of the substantially raised trappin2/elafin production, there may be a paradoxical suppression of cytokine production.

Trappin2/elafin has been measured previously in tissues and CVF from non-pregnant and pregnant women [14]–[15], [34]–[35]. In non-pregnant subjects, high CVF trappin2/elafin concentrations have been associated with greater resistance to HIV infection [34] and in pregnant women trappin2/elafin was found to be increased in amnion from women delivering preterm with chorionamnionitis [14]. These data concur with the suggestion that raised trappin2/elafin rises in a response to subclinical infection/inflammation and processes leading to sPTB. Contrasting reports, have suggested that a failure to induce an appropriate trappin2/elafin response in CVF or the amnion results in bacterial vaginosis [15] and PPROM, respectively [14].

Bastek *et al.* [35], have assessed CVF elafin concentrations in parallel with cervical length and fFN measurements, in pregnant women at 20 weeks' and 23^+6^ weeks' gestation [35]. At these gestations, trappin2/elafin was not useful as a predictor of sPTB. This reinforces our observations that elafin has a much better prediction capacity at 14 weeks', and that this reflects the process of cervical shortening rather than cervical length *per se*.

Comparison of trappin2/elafin between the two studies is not possible as concentrations were not provided by Bastek *et al.* [35], but collection of CVF differed and snap-freezing of swabs in protease inhibitor prior to processing and analysis could release intracellular pre-trappin2 protein due to lysis of cellular material collected on the swab. In contrast, in our study swabs were removed from the protease inhibitor collection media and samples centrifuged to provide a cell free sample prior to storage at −80°C.

A potential limitation of this study, and others, is the use of a commercial human trappin2/elafin ELISA assay, as it is reported to detect the 12.3 kDa (pre-trappin 2) and 9.9 kDa (trappin 2) proteins but is unsuitable for detection of the 6 kD protein (elafin). This would suggest that the raised CVF trappin2/elafin measured in our study reflects an increase in trappin2 specifically. However, using the same antibody, we carried out a pilot study on CVF samples which showed, using western blot, not only bands relating to pre-trappin 2 and trappin 2, but also a band at 6 kDa which approximates the protein size of elafin (data not shown). Future studies should attempt to define the trappin2/elafin ratio in the CVF of women who develop a short cervix, as the proteins can have differing biological properties and excessive neutrophil elastase can reduce tethering of trappin2 to cell membranes and induce proteolytic processing of trappin2 into elafin [29].

Despite sharing similar antimicrobial properties, the CVF cathelicidin profile did not mimic that of trappin2/elafin. The rise in cathelicidin around 18–19 weeks' pregnancy coincided with the mean gestation at which cervical shortening occurred, prompting administration of vaginal progesterone or a cervical suture. As there was no impact of treatment, this increment appeared to be related to the process of cervical shortening. Indeed, cathelicidin is reported in the literature [17] to play a role in wound healing as well as in the innate immune response. The correlation of cathelicidin with CVF cytokines was surprising as *in vitro* cathelicidin is not regulated by cytokines, but implies that the process of cervical shortening does involve both cytokines and cathelicidin.

There are reports to suggest that cathelicidin is regulated by vitamin D *in vitro*, but neither trappin2/elafin or cathelicidin in the CVF was associated with the women's vitamin D status as assessed by measurement of vitamin (OH-25)D. However, this is not unexpected as the majority of women were vitamin D deficient which might obscure any association. It would be of interest to determine whether CVF cathelicidin was induced in a study of vitamin D supplementation.

The clinical importance of this study lies in the identification of CVF elafin/trappin2 as a potentially useful tool to screen women for risk of cervical shortening at a gestational age as early as 14 weeks. Women with raised CVF trappin2/elafin could be stratified to receive more intensive surveillance such as regular cervical length transvaginal ultrasound and, at later gestation, to an fFN test for risk of premature birth. This strategy would optimise clinical resource use and avoid unnecessary surveillance of women unlikely to develop a short cervix on the basis of this new test. Most interventions currently employed to prevent sPTB rely on prior identification of women with a short cervix, generally only identified at later gestations (19–22 weeks') which gives limited time for effective intervention. Early pregnancy recognition of those most likely to develop a short cervix would provide the opportunity for progesterone administration or elective cerclage at an earlier gestation than currently practised. Use of trappin2/elafin as an earlier test of risk of sPTB could similarly identify women suitable for inclusion in clinical trials assessing prophylactic interventions to prevent premature labour.

These observations require prospective validation in other cohorts; we have a study underway to validate trappin2/elafin as a predictor of cervical shortening and sPTB. In parallel, this will enable assessment of CVF and cervical epithelial cell trappin2/elafin concentrations, as well as relationships with neutrophil activity and the CVF microbiome profile.
